# Increased prevalence of overweight and obesity in children with X-linked hypophosphatemia

**DOI:** 10.1530/EC-19-0481

**Published:** 2020-01-07

**Authors:** Volha V Zhukouskaya, Anya Rothenbuhler, Annamaria Colao, Carolina Di Somma, Peter Kamenický, Séverine Trabado, Dominique Prié, Christelle Audrain, Anna Barosi, Christèle Kyheng, Anne-Sophie Lambert, Agnès Linglart

**Affiliations:** 1 APHP, Reference Center for Rare Disorders of the Calcium and Phosphate Metabolism, FilièreOSCAR and Platform of Expertise for Rare Diseases Paris-Saclay, Bicêtre Paris-Saclay Hospital, Le Kremlin-Bicêtre, France; 2 Division of Endocrinology, Department of Clinical Medicine and Surgery, University of Naples Federico II, Naples, Italy; 3 APHP, Department of Endocrinology and Diabetology for Children, Bicêtre Paris Saclay Hospital, Le Kremlin-Bicêtre, France; 4 IRCCS SDN, Naples, Italy; 5 APHP, Department of Endocrinology and Reproductive Diseases, Bicêtre Paris-Saclay Hospital, Le Kremlin-Bicêtre, France; 6 Paris Sud – Paris Saclay University, Faculté de Médecine, Le Kremlin-Bicêtre, France; 7 APHP, Department of Molecular Genetics, Pharmacogenetics and Hormonology, Bicêtre Paris-Saclay Hospital, Le Kremlin-Bicêtre, France; 8 Université Paris V, Faculté de Médecine, Paris, France; 9 Hôpital Necker EnfantsMalades APHP, INSERM U1151, Paris, France; 10 APHP, Department of Adolescent Medicine, Bicêtre Paris Saclay Hospital, Le Kremlin-Bicêtre, France

**Keywords:** X-linked hypophosphatemia, rickets, phosphorus, overweight, obesity

## Abstract

**Background/aim:**

X-linked hypophosphatemia (XLH) is a rare disease characterized by low phosphate levels. Scientific evidence points to a link between hypophosphatemia and obesity in general population. The aim of our longitudinal observational study was to investigate the prevalence of obesity and associated factors in a large cohort of children with XLH.

**Patients/methods:**

We studied 172 XLH-children 5–20 years of age (113 girls/59 boys). Anthropometric parameters (weight, height, and BMI) were collected at birth and during follow-up at mean ages of 5.3, 8.2, 11.3, and 15.9 years (groups 1, 2, 3, and 4, respectively). In each group, subjects were classified based on International Obesity Taskforce (IOTF) cut off values of BMI for age and sex as overweight or obese (IOTF 25–30 or ≥30 kg/m^2^, respectively).

**Results:**

In each age-group, almost 1/3 of XLH-patients were classified as overweight or obese (29.4, 28.7, 27.5, and 36.7% in groups 1, 2, 3, and 4, respectively). Children without a XLH-family history had higher BMI-IOTF at every point of follow-up, compared to those with positive XLH-family history. Similarly, higher BMI-IOTF was significantly associated with treatment duration (23.3 ± 4.4 vs 23.8 ± 3.8 vs 25.2 ± 4.5 kg/m^2^, for subjects with treatment duration of <5, 5–10 and >10 years, respectively, *P* for trend = 0.025). Multiple regression analysis confirmed an association of treatment duration and lack of XLH-family history with higher BMI-IOTF.

**Conclusion:**

One out of three of XLH-children have phenotypically unfavourable metabolic profile expressed as increased prevalence of overweight or obesity in comparison to general population. Both the lack of XLH family history and the duration of treatment increase the risk of higher BMI-IOTF. BMI should be carefully monitored in children, and later in adults, with XLH.

## Introduction

X-linked hypophosphatemia (XLH) is a rare disease caused by inactivating mutations in the phosphate-regulating endopeptidase homolog X-linked (*PHEX*) gene and characterized by chronic hypophosphatemia. Impaired function of *PHEX* leads to the upregulation of the expression of phosphaturic fibroblast growth factor 23 (FGF23) in bone, which is secreted in the plasma and induces renal phosphate-wasting hypophosphatemia and low levels of calcitriol (1,25(OH)_2_D) *via* inhibition of 1α-hydroxylase and activation of 24-hydroxylase. Clinically, XLH children are characterized by progressive skeletal deformities (leg bowing, waddling gait, poor growth, and disproportional short stature), dental abscesses, craniosynostosis, and typical radiographic changes of rickets (1). Current medical treatment consisting of oral active vitamin D (calcitriol or 1α-(OH)D_3_) and multiple daily phosphate supplementation partially restores clinical, biochemical, and radiographic signs of rickets; however, it is not able to rescue phosphate wasting and maintain normal levels of serum phosphate (2). Thus, patients live with chronic low levels of phosphate, and consequences of this are currently poorly studied.

It has recently been hypothesized that low phosphorus intake may be involved in the progression of weight gain and metabolic syndrome (3, 4, 5, 6, 7, 8, 9, 10, 11). Today, a growing body of scientific evidence shows an inverse relationship between serum phosphate level and BMI. On the other hand, phosphorus supplementation for 3 months significantly decreased body weight, BMI, waist circumference; restored diet-induced thermogenesis; and increased post-prandial satiety in obese/overweight subjects (10, 11).

Based on the putative role of phosphate in metabolic syndrome and the permanent abnormal phosphate level observed in XLH children, we performed a longitudinal observational study to investigate the anthropometric parameters of overweight and obesity and its evolution throughout time in a large cohort of children affected by XLH.

## Methods

### Study design and patients

The present study is a retrospective longitudinal observational study which was conducted in our Reference Center for Rare Disorders of the Calcium and Phosphate Metabolism, Filiere OSCAR and Platform of expertise for rare diseases Paris-Saclay, Bicêtre Paris-Saclay Hospital, France, where 263 patients (adults and children) with XLH are registered. In this center, the diagnosis of XLH is made on the basis of clinical (family history, symptoms, and physical examination), biochemical (hypophosphatemia, renal phosphate wasting), and molecular analysis (mutation in the *PHEX* gene) criteria. As soon as a child is diagnosed with XLH, we perform an examination of parents too which comprises of a detailed clinical history focused on possible XLH manifestations during childhood such as leg deformities, recurrent dental abscesses, poor growth, short stature, and pain; a measurement of serum phosphate level; and genetic analysis for the *PHEX* gene mutation. The absence of clinical, biochemical signs of hypophosphatemia in association with negative genetic analysis for the *PHEX* mutation indicates the absence of XLH in parents. Therefore, in this case we safely conclude for the lack of family history.

We selected only children with XLH who had minimum follow-up of 3 years from diagnosis. The exclusion criteria were the lack of follow-up data; children born preterm (less than 36 weeks of gestation); and children who received treatment with recombinant human growth hormone (rhGH) and/or with a monoclonal antibody anti-FGF23 (burosumab) (we excluded the follow-up period during and after treatment with rhGH and/or burosumab). Data recorded during conventional therapy, that is, active vitamin D analogues (alfacalcidol) and phosphate supplements, were included. According to these inclusion and exclusion criteria, 172 children affected with XLH were included in the present study.

As soon as diagnosis was completed, all patients started treatment with active vitamin D analogues (alfacalcidol) and phosphate supplements according to recommendations (2, 12) and continued treatment during the follow-up period.

According to the Jardé law in France, the study was approved by the French National Data Processing and Liberties Commission (CNIL). The need for written consent is therefore waived by this law. Patients and/or their parents were informed orally of the content of the study, and their consent was obtained. They have the right to claim their opposition (i.e. refuse to participate) to the study at any time by sending a correspondence to http://recherche.aphp.fr/eds/droit-opposition.

### Study protocol and methods

The entire patients’ follow-up period was from birth till the age of 20 years and was divided into five sub-periods defining age groups from 0 to 4. Group 0 included the parameters at birth, and groups 1, 2, 3, and 4 comprised the follow-up parameters in age period of 5–7 years, of 7–10 years, of 10–15 years, and of 15–20 years, respectively. Since there are a lot of changes occurring in the first years following the start of therapy, including auxology and mineral metabolism, we thought that 5-year intervals may lack precision in this matter and have reduced the initial intervals in groups of 5–7 years and 7–10 years. For each patient, we gathered the data for each age group and these data were gestational age and auxological parameters such as weight, length/height, and BMI. Most of the patients had follow-up parameters available for all age groups; however, some of them had incomplete data, for example, follow-up data only till the age of 10 years or follow-up data only for age groups 0, 1, and 3 or 0, 2, and 4. Therefore, we recorded the follow-up parameters for 126 patients in age group 1 (5–7 years), for 88 patients in age group 2 (7–10 years), for 91 patients in age group 3 (10–15 years), and for 49 patients in age group 4 (15–20 years). Patients were followed in only one center by AR, PK, ASL, or AL.

BMI was calculated using the formula weight (kg)/height (m)^2^. Z-scores (SDS) for length/height, weight, and BMI were derived from the World Health Organization (WHO) growth charts (13). Z-score for weight was obtained only for children from 0 to 10 years. In each group, subjects were classified based on International Obesity Taskforce (IOTF) BMI cut off values for age and sex as overweight or obese (BMI IOTF 25–30 or ≥30 kg/m^2^, respectively) (14).

Intact FGF23 was measured on fasting samples. Intact FGF23 measurements were performed since 2006 at Hôpital Necker Enfants Malades APHP, INSERM U1151, Paris, France. Until 2017, intact FGF23 was measured by a two-site ELISA (Immunotopic, San Clemente, CA, USA). The intra-assay coefficients of variation were respectively 4.4 and 2.6% at a concentration of 14.6 and 148 ng/L. The inter-assay coefficients of variation were respectively 6.1 and 6.5% at a concentration of 15.6 and 166 ng/L. The detection limit was 1.0 ng/L. Since 2017, FGF23 levels have been measured through a new automated immuno-chemiluminescent sandwich assay (DiaSorin, Saluggia, Italy) on the Liaison XL platform. Intra-assay coefficients of variation were 2.86 and 2.33% at 50.5 and 242.3 ng/L, respectively. Inter-assay coefficients of variations were 6.3 and 2.1% at 34.6 and 123.7 ng/L, respectively. The detection limit was 10.0 ng/L.

In our centre, the genetic analysis for *PHEX* mutation is performed through gene panel analysis using next-generation sequencing. All our patients who do not bear a *PHEX* mutation have a disease inheritance pattern that is compatible with a X-linked disease inheritance pattern.

### Statistical analysis

Statistical analysis was performed by IBM SPSS Statistics for Windows, version 25.0 base (IBM Corp.). The results are expressed as mean ± s.d. and median (Lower Quartile (LQ) – Upper Quartile (UQ)) for continuous variables and percentage with absolute number in parenthesis for categorical variables. The comparison of continuous variables were performed using Student’s *t* test or Mann–Whitney *U* test or by one-way ANOVA analysis, as appropriate. Categorical variables were compared by χ^2^ test or Fisher’s exact test, as appropriate. Multivariate linear regression analysis assessed the association between BMI-IOTF (dependent variable) and the independent variables such as duration of treatment with phosphorus supplements (expressed as categorical ordinal variable: less than 5 years, 5–10 years and more than 10 years) and XLH-family history (expressed as categorical nominal variable: presence or absence). These variables were chosen since they were found to be associated with BMI-IOTF in correlation analysis and one-way ANOVA. Since BMI-IOTF is already adjusted by sex and age, therefore, the multiply regression analysis does not require further adjustment for these parameters. *P* values of less than 0.05 were considered significant.

Figures were performed with GraphPad Prism for Windows, version 7.0.

## Results

### Characteristics of the cohort of children with XLH

The general characteristic of 172 children affected by XLH is described in Table 1. The average age of patients in age groups 1, 2, 3, and 4 was 5.3 ± 0.5, 8.2 ± 1.0, 11.3 ± 1.4, and 15.9 ± 1.0 years, respectively. There were no differences in the distribution of boys and girls between age groups (Table 2).

**Table 1:** Description of the cohort of children affected by X-linked hypophosphatemia.

Parameter	Absolute number or % (*n*) or mean ± s.d. and median (LQ-UQ)
Number of subjects	172
Boys/girls	34.3 (59)/65.7 (113)
Subjects carrying a *PHEX*-mutation	88.4 (130)
Subjects with positive XLH-family history:	59.7 (92)
– father affected by XLH – mother affected by XLH	27.2 (25) 72.8 (67)
Diagnosis of XLH, years	3.0 ± 2.9 2.0 (1.0–3.3)
– <1 year of age – 1 –5 years of age – 5 –10 years of age – >10 years of age	0.6 ± 0.3 2.4 ± 0.8 6.6 ± 1.4 12.1 ± 2.3
Number of subjects diagnosed with XLH at age:	
– <1 year of age – 1 –5 years of age – 5 –10 years of age – >10 years of age	26.6 (41) 55.8 (86) 12.3 (19) 5.2 (8)
Duration of follow-up, years	10.9 ± 4.0 10.2 (7.4–15.0)
Gestational age, weeks	39.0 ± 1.3 39 (38–40)
Birth weight, kg	3.3 ± 0.5 3.3 (3.0–3.7)
Birth weight, SDS	0.0 ± 0.0 0.0 (−0.6 to 0.7)
Birth length, cm	49.5 ± 2.1 50.0 (48.0–51.0)
Birth length, SDS	0.4 ± 4.2 0.1 (−0.6–0.9)
Subjects born SGA	6.9 (9)

BMI, body mass index; LQ, lower quartile; SGA, small for gestational age; UQ, upper quartile; XLH, X-linked hypophosphatemia.

**Table 2:** Postnatal anthropometric parameters of obesity in different age groups of children affected with X-linked hypophosphatemia.

Parameter	Group 1 (5–7 years)	Group 2 (7–10 years)	Group 3 (10–15 years)	Group 4 (15–20 years)	*P*
Boys + girls					
Number of subjects	126	88	91	49	–
Age at evaluation, years	5.3 ± 0.5 5.2 (5.0–5.6)	8.2 ± 1.0 8.2 (7.3–9.0)	11.3 ± 1.4 10.7 (10.2–12.0)	15.9 ± 1.0 15.9 (15.3–16.4)	–
Boys/girls, % (*n*)	34.9 (44)/65.1 (82)	31.8 (28)/68.2 (60)	33.0 (30)/67.0(61)	30.6 (15)/69.4 (34)	0.94
BMI, SDS	0.9 ± 1.0 0.8 (0.3–1.4)	0.8 ± 1.0 0.6 (0.1–1.3)	0.6 ± 1.1 0.6 (−0.1 to 1.4)	0.7 ± 1.0 0.9 (0.0–1.5)	0.35
BMI-IOTF, kg/m^2^	23.5 ± 4.3 23.2 (20.6–25.4)	23.5 ± 4.0 22.5 (21.0–25.4)	23.7 ± 4.2 23.0 (20.7–25.4)	24.3 ± 3.6 24.1 (21.6–26.9)	0.68
Number of subjects, % (*n*):					
– overweight – obese	21.4 (27) 7.9 (10)	19.5 (17) 9.2 (8)	16.5(15) 11 (10)	30.6 (15) 6.1 (3)	0.60
Boys					
Boys BMI, SDS	1.0 ± 1.2 1.0 (0.2–1.7)	1.0 ± 0.9 0.9 (0.4–1.7)	0.8 ± 0.9 0.9 (0.1–1.4)	0.55 ± 1.0 0.9 (−0.3 to 1.2)	1.0
Boys BMI-IOTF, kg/m^2^	23.3 ± 4.8 23.1 (20. –25.5)	23.9 ± 2.8 23.8 (21.4–26.4)	23.6 ± 3.2 23.2 (21.4–25.2)	23.5 ± 3.3 24.1 (20.8–25.7)	0.93
Number of subjects, % (*n*):					
– overweight – obese	25.0 (11) 9.1 (4)	37.0 (10) 3.7 (1)	20 (6) 6.7 (2)	13.3 (2) 13.3 (2)	0.61
Girls					
Girls BMI, SDS	0.85 ± 0.9 0.8 (0.4–1.3)	0.65 ± 1.0 0.6 (0.1–1.1)	0.56 ± 1.2 0.3 (−0.3 to 1.3)	0.8 ± 1.2 0.7 (0.1–1.7)	0.82
Girls BMI-IOTF, kg/m^2^	23.6 ± 4.1 23.3 (20.8–25.2)	23.3 ± 4.5 21.9 (20.6–24.7)	23.7 ± 4.6 22.1 (20.3–25.4)	24.6 ± 3.7 23.7 (22.0–27.8)	0.59
Number of subjects, % (*n*):					
– overweight – obese	19.5 (16) 7.3 (6)	11.7 (7) 11.7 (7)	14.8 (9) 13.1 (8)	38.2 (13) 2.9 (1)	0.042

The results are expressed as mean ± s.d. and median (Lower Quartile – Upper Quartile) or percentage with absolute number in parenthesis.

BMI-IOTF, Body Mass Index – International Obesity Task Force.

In the entire cohort of 172 patients with XLH, the information about genetic analysis was available for 147 patients among which 130 patients had a mutation in their *PHEX* gene (88%); in 17 patients (12%), we did not find a mutation. We were not able to retrieve any information about genetic analysis for 25 patients who were diagnosed with XLH in other centres in France and who were referred to our center for follow-up later on. Sixty percent (*n* = 92) have a positive family history for XLH, of which 2/3 of patients were born to XLH affected mothers. Intact FGF23 was on average equal to 279.3 ± 277.3 ng/L. As expected from X-linked dominant inheritance, two-third of the patients were girls (65.7%) and one-third were boys (34.3%). In this monocentric cohort, the mean age of XLH diagnosis and start of treatment for the whole cohort was 3.0 ± 2.9 years. One half of the children (55.8%, *n* = 86) were diagnosed with XLH at a mean age of 2.4 ± 0.8 years. One-third of the children (26.6%, *n* = 41) had early diagnosis of XLH due to already known family history (0.6 ± 0.3 years). Nineteen patients were diagnosed with XLH in the age period between 5 and 10 years (mean age 6.6 ± 1.4 years) and 8 patients in the age period after 10 years (mean age 12.1 ± 2.3 years). The average duration of follow-up was 10.9 ± 4.0 years (Table 1).

### Anthropometric parameters of neonates and children with XLH

The average birth weight and length of children with XLH was 3.3 ± 0.5 kg (0.0 ± 0.0 SDS) and 49.5 ± 2.1 cm (0.4 ± 4.2 SDS), respectively. In our cohort of children affected by XLH, 6.9% (*n* = 9) of them were considered at birth as small for gestational age (weight and/or length < − SDS than expected for their gestational age or weight <2500 g in presence of unknown gestational age) (15, 16) (Table 1). Children without a XLH-family history had a higher birth length (50.0 ± 1.8 cm (1.3 ± 6.4SDS) vs 49.0 ± 2.3 cm (−0.3 ± 1.2SDS), *P* = 0.006, respectively) and tended to have a lower prevalence of SGA compared to those with XLH-family history (3.6% vs 8.7%, *P* = 0.3, respectively). Regarding the children with a positive XLH-family history, neonates born from affected mother had lower birth weight (3.2 ± 0.5 kg (−0.3 ± 1.0 SDS) vs 3.5 ± 0.5 kg (0.5 ± 1.0 SDS), *P* = 0.031 and 0.008, respectively) and higher prevalence of SGA (12.2% vs 0%, *P* = 0.17, respectively) compared to those who had father affected with XLH.

**Figure 1:**
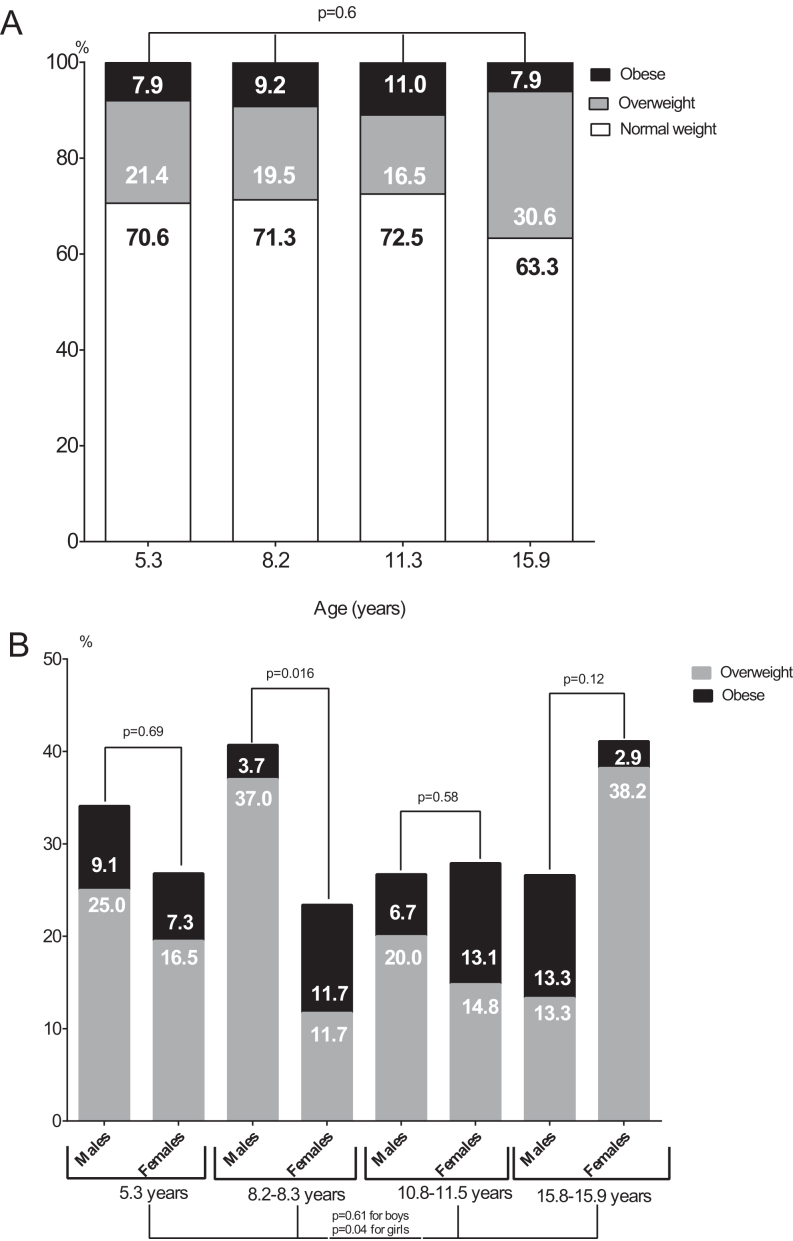
Prevalence of overweight and obesity in the different age-groups (A) of children affected by X-linked hypophosphatemia, further divided by sex (B).

Anthropometric parameters of XLH children in different age group are described in Table 2. Overall, one-third of XLH children were overweight or obese in each age group. Although without reaching statistical significance, a trend of increasing prevalence of overweight/obese subjects overtime with age was noticed, especially in age group 4 (29.4, 28.7, 27.5, and 36.7% for age groups from 1 to 4, respectively, *P* = 0.6) (Fig. 1A and Table 2).

When considering gender, the progression of postnatal anthropometric parameters followed different pattern in girls and boys (Fig. 1B and Table 2). Girls affected by XLH showed a statistically significant increase of overweight/obese patients from age groups 1 to 4, with the highest prevalence of 41.1% in the oldest age group. Boys affected by XLH showed the highest prevalence of overweight/obese subjects of 40.7% in age group 2. After the age of 10 years, there was a decrease of overweight/obese subjects to 27% among boys affected by XLH. 

**Figure 2:**
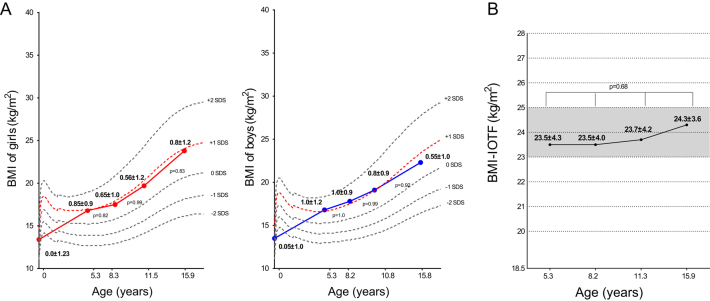
Progression of postnatal BMI-SDS divided by sex (A) and BMI-IOTF (B) in children affected by X-linked hypophosphatemia. BMI-IOTF, Body Mass Index – International Obesity Task Force.

The progression of postnatal anthropometric parameters in terms of BMI-SDS and BMI-IOTF is presented in Fig. 2A, B and Table 2. Both girls and boys showed a similar pattern of BMI-SDS progression close to +1.0 SDS over time (Fig. 2A). BMI-IOTF tended to increase over time from 23.5 ± 4.3 in age group 1 to 24.3 ± 3.6 kg/m^2^ in age group 4, without reaching statistical significance, nonetheless (*P* = 0.68) (Fig. 2B).

A different pattern of BMI-SDS progression was noticed according to the age of start of phosphorus supplements. Children who started the treatment at 2.4 ± 0.8 years had BMI-SDS +1.1 ± 1.0 before starting the treatment. After starting phosphorus supplements, BMI-SDS progressively decreased, reaching 0.7 ± 1.0 SDS at the age of 15 years. Children who were treated with phosphorus supplements since birth maintained normal BMI-SDS (0.6 ± 0.8) till age between 5–7 years. Thereafter, BMI-SDS increased and remained stable at +1.0 SDS in age groups 2, 3, and 4.

### Clinical factors associated with overweight and obesity in children with XLH

Considering an elevated prevalence of overweight/obese subjects in our cohort of children affected with XLH, we looked for clinical factors likely to be associated with the increased weight gain in this population.

#### XLH-family history

**Figure 3:**
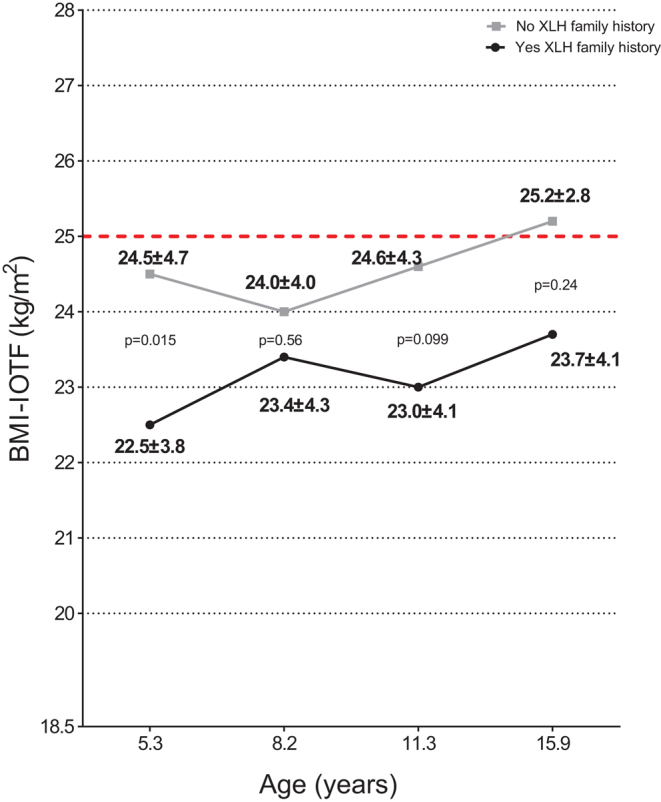
Progression of postnatal BMI-IOTF according to XLH-family history in children affected by X-linked hypophosphatemia. BMI-IOTF, Body Mass Index – International Obesity Task Force; XLH, X-linked hypophosphatemia.

Since we identified the differences in the auxological parameters according to XLH-family history, we studied BMI-IOTF in children with and without XLH-family history. BMI-IOTF increased over time in both groups of children (Fig. 3). However, patients without a XLH-family history had higher BMI-IOTF in all age groups compared to those with a positive XLH-family history. No significant differences in BMI-IOTF were found overtime between children born from mother or father with XLH (data not shown).

#### Duration of treatment with phosphorus supplements

Taking in account an increase of BMI-SDS after about 7–10 years of treatment with phosphorus supplements in patients who started the treatment since birth, we divided the patients in groups according to treatment duration: <5 years, 5–10 years, and >10 years.

**Figure 4:**
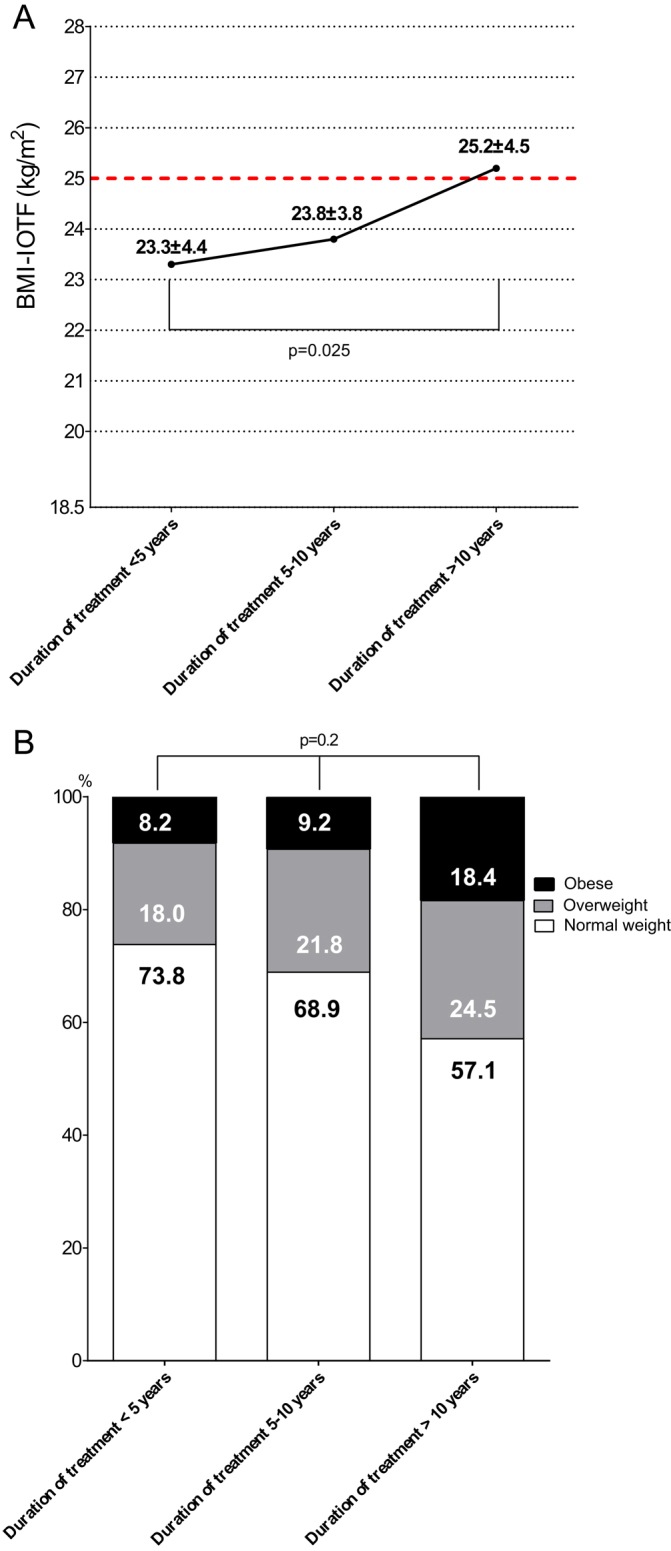
Progression of postnatal BMI-IOTF (A) and the prevalence of overweight and obesity (B) according to the duration of treatment with phosphorus supplements in children affected by X-linked hypophosphatemia. BMI-IOTF, Body Mass Index – International Obesity Task Force.

BMI-IOTF significantly increased with duration treatment (23.3 ± 4.4 vs 25.2 ± 4.5 kg/m^2^ for those with treatment duration <5 years and >10 years, respectively, *P* = 0.025) (Fig. 4A). Patients who were treated with phosphate supplements for more than 10 years had higher prevalence of overweight/obesity (42.9%), compared to those who were treated for less than 5 years (20.2%, *P* = 0.2) (Fig. 4B).

#### Association of BMI-IOTF with XLH-family history and duration of treatment with phosphorus supplements

Multiply regression analysis confirmed the significant association between higher BMI-IOTF and the lack of XLH-family history (β = −0.13, 95%CI = −0.12 to −2.21, *P* = 0.029) and higher duration of treatment with phosphorus supplements (β = 0.17, 95%CI = 0.30-1.73, *P* = 0.005) in children affected by XLH.

## Discussion

The present longitudinal observational study is the first to show an elevated prevalence of overweight or obesity in children affected by XLH. In particular, we described that their BMI rises early in life with a progressive increase over-time, especially in adolescent girls. We identified factors associated with higher BMI such as a lack of XLH-family history and a longer duration of phosphorus supplements.

Obesity represents a worldwide problem. Over the past three and half decades, the prevalence of obesity has nearly doubled worldwide. Among adults aged 18 years or older, 11% of men and 15% of women were obese in 2014, and more than 42 million children under the age of 5 years were overweight in 2013 (17). In France, 20% of children were overweight or obese in 2013 (18).

In our study, we found that almost 30% of children affected by XLH are overweight or obese in every age group, which is higher in comparison to French pediatric population (18). In our cohort, overweight/obesity progresses with age, considering that at the age of 15 years, adolescents with XLH had higher BMI-IOTF (Fig. 2B) and higher prevalence of overweight or obesity of 37%, in comparison to other age groups (Fig. 1A). Additionally, we noticed a different pattern of progression of overweight/obesity between boys and girls. Boys affected by XLH were significantly overweight/obese in ages of 7–10 years (40.7%), and girls affected by XLH had the highest prevalence of overweight/obesity after the age of 15 years (Fig. 1B).

Higher BMI in children affected by XLH shown in this study is in accordance to other studies which showed an inverse relationship between serum phosphate level and BMI (3, 4, 5, 6, 7, 8, 9, 10, 11). From pathophysiological point of view, the possible explanation of the link between phosphorus and weight gain resides in ATP production which depends upon adequate sources of phosphorus. First, decline of ATP production, especially at hepatic level, due to hypophosphatemia, transduces the signals to the CNS which leads to increased food intake. Secondly, low ATP production decreases thermogenesis and energy expenditure (3). Thus, this is a hypothesis on how low phosphorus status may contribute to the development of obesity through the regulation of food intake, thermogenesis, and energy expenditure. In support to this, phosphorus supplementation for 3 months is able to significantly reduce body weight, BMI, waist circumference (10), to restore diet-induced thermogenesis, and to increase post-prandial satiety (11) in obese/overweight subjects. Animal models (19) demonstrated that high-phosphate diet suppresses the activity of white adipose tissue by increasing lipolytic and decreasing lipogenic gene expression. In other words, state of chronic hypophosphatemia would sustain the weight gain, and vice versa, phosphate supplementation would help to lose the weight.

Our results are in accordance with that. Indeed, the children who were diagnosed with XLH in period between ages of 1–5 years, had BMI-SDS already above +1.0 SDS at the moment of diagnosis before starting appropriate treatment. After starting the treatment with phosphorus supplements, BMI-SDS decreased overtime. Taking in consideration a positive effect of phosphate treatment on weight loss, one would expect much lower prevalence of overweight or obesity in XLH children under the therapy. In contrary, we found an increased prevalence of overweight/obesity in children affected by XLH in each age group, despite the appropriate therapy. Regardless of the possible protective role of phosphate supplements at the treatment initiation, this therapy is not able to prevent weight gain over time in the context of chronic hypophosphatemia in XLH. It may happen due to different reasons. First, although phosphate supplementation corrects clinical, biochemical, and radiographic signs of rickets, it is not able to maintain a stable level of serum phosphate (2). Thus, one cannot exclude the persistent hypophosphatemia during the day, despite multiply daily doses. Secondly, it should be taken in an account that the concomitant presence of skeletal abnormalities also requires the adequate level of serum phosphorus to be healed. Therefore, the doses of phosphate supplementation used in XLH are probably not sufficient to heal bone lesions and to prevent weight gain.

A surprising result of our research is the positive association of BMI-IOTF with the duration of treatment. After 10 years of treatment with phosphate, a significant increase of BMI-IOTF and of the prevalence of overweight/obesity was seen in children with XLH (Fig. 4A and B). Probably, this association would indirectly suggest that this kind of therapy loses its protective effect from weight gain after 10 year of its usage. Unfortunately, there are neither clinical nor basic studies till now that could give an appropriate explanation for this association, and our study is the first one to show it. It is possible that the phosphate supplementation at the beginning of treatment could be sufficient for all body’s needs (skeletal and extra-skeletal). However, some period of rapidly growing skeleton, such as puberty, requires more phosphorus to build the bone leaving lesser amount of phosphorus for the extra-skeletal body’s needs and, consequently, to the loss of protective effect of phosphate supplementation from weight gain. This is our speculation that should be further studied and confirmed with basic and clinical studies. Nonetheless, length of treatment for more than 10 years should be considered as a ’surrogate marker’ of increased risk of higher BMI-IOTF in XLH-children.

Another interesting result of our study is the association of BMI-IOTF with the lack of XLH-family history. Unfortunately, no study so far, neither animal nor human, addressed the link between obesity and genetic background in XLH. Nonetheless, more attention to weight control should be payed to the children affected by XLH with *de novo PHEX* mutation.

The present study has several limitations. First, we did not include a control group in the study. Nonetheless, comparing to the recently published data on the prevalence of overweight/obesity among French pediatric population (18), children with XLH are more overweight or obese. Secondly, only children were included for follow-up evaluation. Further studies on adult population are needed in order to investigate and confirm an increased prevalence of overweight/obesity in adult patients with XLH. Thirdly, BMI is a crude parameter of obesity which cannot distinguish the adipose tissue from muscular and other tissues. Therefore, Dual-Energy X-ray Absorptimetry should further be used to better characterize the body composition in patients with XLH and to confirm our findings. Finally, we did not take into account a family history of obesity, dietary habits, daily caloric consumption, and physical activity in children with XLH to exclude the confounding factors.

However, the biggest strength of our study is its longitudinal design and evaluation on the large and homogeneous cohort of patients. Moreover, since our centre represents the Reference Center for Rare Disorders of the Calcium and Phosphate Metabolism and it collects the patients from all over France, we therefore safely conclude that the cohort of participants is a true reflection of French population.

The results of our study have an important impact for health-care providers. In clinical routine practice, careful evaluation and consistent monitoring of BMI are required for children affected by XLH. It may be of utmost importance for adulthood, given the risk for rheumatologic complications in adults with XLH. Finally, the lack of XLH-family history and longer duration of phosphate supplementation could help to individuate those patients for whom a particular attention should be given in controlling the weight gain.

In conclusion, one-third of XLH-children have phenotypically unfavourable metabolic profile expressed as overweight or obesity. Lack of XLH family history and length of treatment increase the risk of higher BMI-IOTF.

## Declaration of interest

The authors declare that there is no conflict of interest that could be perceived as prejudicing the impartiality of the research reported.

## Funding

This research did not receive any specific grant from any funding agency in the public, commercial, or not-for-profit sector.

## Data availability

The datasets generated and analysed during the current study are not publicly available but are available from the corresponding author on reasonable request.
